# Comparative Investigation of Antimicrobial and Antioxidant Effects of the Extracts from the Inflorescences and Leaves of the *Cannabis sativa* L. cv. *strawberry*

**DOI:** 10.3390/antiox12020219

**Published:** 2023-01-18

**Authors:** Laura Serventi, Giancarlo Angeles Flores, Gaia Cusumano, Davide Barbaro, Bruno Tirillini, Roberto Venanzoni, Paola Angelini, Alessandra Acquaviva, Simonetta Cristina Di Simone, Giustino Orlando, Gokhan Zengin, Luigi Menghini, Claudio Ferrante

**Affiliations:** 1 Department of Chemistry, Biology and Biotechnology, University of Perugia, 06122 Perugia, Italy; 2 Botanic Garden “Giardino dei Semplici”, Department of Pharmacy, “Gabriele d’Annunzio” University, Via dei Vestini 31, 66100 Chieti, Italy; 3 Department of Biomolecular Sciences, University of Urbino, 61029 Urbino, Italy; 4 Physiology and Biochemistry Research Laboratory, Department of Biology, Science Faculty, Selcuk University, 42130 Konya, Turkey

**Keywords:** *Cannabis sativa*, antimicrobial, antioxidant, phenolic compounds, benzoic acid, cannabidiol, cannabidiolic acid

## Abstract

*Cannabis sativa* products have historically been used for healing purposes; now their biological properties are supported with scientific evidence, but modern research has not yet fully developed its therapeutic potential. This study focuses on the cultivar of *C. sativa* called strawberry to understand the biological and medical potentials of hydroalcoholic extracts from two different parts of the plant: leaves and inflorescences. Two biological assets were investigated including antioxidant and antimicrobial potential. Additionally, quantitative determination of phenolic and terpenophenol compounds was conducted. The antimicrobial action was highlighted for the hydroalcoholic extract from inflorescences, especially against *Escherichia coli* and *Bacillus subtilis.* Among the dermatophytes’ strains, the most sensitive was *Arthroderma currey*. These effects could be related albeit partially to the pattern of the phenolics detected, among which the most prominent one was benzoic acid. On the other hand, antioxidant and antimicrobial effects of the extracts could be also mediated by the main terpenophenolics identified and quantified, namely cannabidiolic acid and cannabidiol. Collectively, the present data point to the potential use of the inflorescences from the *C. sativa* cultivar strawberry as a valuable plant material for the development of bioactive extracts with antioxidant and antimicrobial effects

## 1. Introduction

Plants have long been used for health-promoting effects, due to the bioactivity of specialized metabolites that may work as medicines, flavorings, and recreational substances in humans [1,2,3,4].

In this context, a great deal of attention has been paid to *Cannabis sativa* L. [5], an annual, robust, fast-growing, and generally dioecious plant that produces male and female flowers on separate individuals, although it has the tendency to be monoecious [6,7]. 

Industrial hemp has been long cultivated as a valuable source of fibers and nutrients [5], and numerous European countries, including Italy, have promoted the valorization of the hemp productive chain, thus driving the registration of new cultivars, with more than 60, throughout Europe. This market is currently ruled by the EU (Regulation (EC) N° 1251/99 and subsequent amendments), according to which only hemp cultivars registered can be cultivated and after the verification of Δ9-tetrahydrocannabinol content, which has to be lower than 0.2% *w/w* [8].

Non-psychotropic terpenophenols, cannabidiol and cannabigerol, are the main phytochemicals responsible for hemp’s pharmacological effects, especially in the brain [9,10,11,12,13]. On the other hand, specialized metabolites present in trichomes, including terpenes and phenolic compounds, may influence the biological properties of inflorescence extracts [14,15,16,17,18]. 

Previous studies have suggested the potential pharmacological applications of polar extracts from inflorescences, with antioxidant/anti-inflammatory and antimicrobial properties related, albeit partially, to the pattern of phenolic compounds [19,20]. In addition, the aerial parts of the plant such as the leaves are rich in trichomes thus suggesting that leaves also contain active ingredients and their biological activities could be investigated.

Actually, limited data are available about the potential applicability of hemp leaves as a source of bioactive extracts with health-promoting effects [21]. Therefore, the objective of this study was to investigate the antimicrobial and antioxidant activity of hydroalcoholic extracts from inflorescences and leaves of the *C. sativa* strawberry cultivar. The extracts were prepared via ultrasound-assisted extraction (UAE) and analyzed through liquid chromatography for the quantitative determination of phenolic and terpenophenolic compounds.

## 2. Materials and Methods

### 2.1. Hemp Material

Hemp dry leaves and inflorescences of *Cannabis sativa* L. cultivar “strawberry” were cultivated in the Umbria region, Italy. All of the samples were kindly supplied by J.j. Farm Società Agricola Semplice (Castiglione Del Lago (PG), Italy) during the cultivation season of 2021. Morphological identification was made by Prof. Paola Angelini, Associate Professor at the Department of Chemistry, Biology and Biotechnology, Università degli Studi di Perugia, Perugia (Italy). 

### 2.2. Chemicals and Reagents

1,1-Diphenyl-2-picryl-hydrazyl-hydrate (DPPH), Trolox, 2,4,6-tri(2-pyridyl)-1,5,5-triazine ligand (TPTZ), 2,2′-Azino-bis (3-ethylbenzothiazoline-6-sulfonic acid) or ABTS, ferric chloride, acetate buffer, Mueller–Hinton broth (MHB), Rose bengal chloramphenicol agar (RBCA), malt extract agar (MEA), Sabouraud dextrose agar (SDA), RPMI (Roswell Park Memorial Institute) 1640 medium, Morpholinepropanesulphonic acid (MOPS), fluconazole, and purity grade organic solvent ethanol absolute were purchased from Sigma (Sigma-Aldrich GmbH, Taufkirchen, Germany). 

### 2.3. Molecular Identification

To test our identification based on the morphological characters of *C. sativa*, total genomic DNA was extracted using a ZR plant/seed DNA kit (Euroclone S.p.A., Milan, Italy). The genomic DNA’s quality and quantity were evaluated according to the literature [22]. 

### 2.4. Preparation of Plant Extracts

Female *Cannabis* flowers and leaves were weighed; after, they were gently crushed into small-sized pieces and they were placed into different flasks. To extract the active ingredients, maceration of the plant’s parts in aqueous and hydroalcoholic solutions is required [23], according to Salhi et al. [24] The optimal ratio is 1:10 w/v. Briefly, hydroalcoholic extracts (70% ethanol) were obtained by soaking the flowers and leaves in different bottles with distilled water. The samples were taken into a dark place at room temperature (21 ± 2 °C) for 72 h. According to Abubakar and Haque [25] the extraction was carried out by placing flasks in an ultrasonic bath (Bransonic, Dietzenbach, Germany) for 2 h for each sample. The solutions were filtered using filter paper into clean flasks. Each extract was filtered using a syringe filter with a 0.45 μm pore Ø, bought by Corning (Wiesbaden, Germany). The sterilized extracts were stored at −20 °C. Ten mL of each extract was brought to dryness in the oven at 60 °C until a constant mass was obtained; the dry weight was found and used to calculate the concentration (Table 1).

### 2.5. Antimicrobial Tests 

#### Fungal and Bacterial Strains 

Extracts E1–E2 were tested for in vitro antifungal activity against different yeasts and dermatophyte species: *Candida albicans* (YEPGA 6183), *C. tropicalis* (YEPGA 6184), *C. albicans* (YEPGA 6379), *C. parapsilopsis* (YEPGA 6551), *Arthroderma curreyi* (CCF 5207), *A. gypseum* (CCF 6261), *A. insingulare* (CCF 5417), *A. quadrifidum* (CCF 5792), *Trichophyton mentagrophytes* (CCF 4823), *T. mentagrophytes* (CCF 5930), *T. rubrum* (CCF 4933), and *T. tonsurans* (CCF 4834).

Furthermore, the same samples were assayed for the antimicrobial test against Gram-negative bacterial strains: *Escherichia coli* (ATCC 10536), *E. coli* (PeruMycA 2), *E. coli* (PeruMycA 3), *Pseudomonas aeruginosa* (ATCC 15442), and *Salmonella typhy* (PeruMyc 7), and also against Gram-positive strains: *Bacillus cereus* (PeruMycA 4), *B. subtilis* (PeruMyc 6), and *Staphylococcus aureus* (ATCC 6538). The microbial cultures were maintained in the culture collection of the Department of Chemistry, Biology and Biotechnology (DCBB University of Perugia, Perugia, Italy) named “PeruMyc” and are available upon request. 

Antifungal and antibacterial activities were assessed as previously reported [19,26,27,28].

### 2.6. Antioxidant Tests and Determination of Phenolic Compounds 

The scavenging/reducing effects of the extracts were evaluated through DPPH, ABTS, and FRAP assays. The detailed protocols are reported in the literature [29,30,31,32].

The total amount of phenolic compounds content in *C. sativa* strawberry extracts was determined according to the Folin–Ciocalteu assay [33]. 

### 2.7. High-Performance Liquid Chromatography (HPLC) Analysis of Phenolic Compounds

The extracts were analyzed for phenol and terpenophenol quantitative determination using a reversed phase HPLC-DAD in gradient elution mode [16]. The separation is fully detailed in the Supplementary Materials. 

## 3. Results and Discussion

### 3.1. Plant Identification

Recently, there has been renewed global interest in the therapeutic potential of *Cannabis*, given its unique chemical components. The characterization and identification of this medicinal plant is fundamental for the knowledge of all of its phytochemistry, in such a way as to express its full potential for pharmaceutical applications [34]. The morphological characteristics of *C. sativa* correspond to those reported by Small [11]. The taxonomic affiliation of the plant was performed via targeting the trnL-F region of the chloroplast genome. Additionally, BLAST research (Table 2) has highlighted that the highest omology was observed with the strains cv. Dagestani, Cheungsam, Yoruba, and Carmagnola.

### 3.2. Antimicrobial Activity

Table 3, Table 4 and Table 5 shows the MIC range and geometric means of cv strawberry extracts and synthetic drugs (ciprofloxacin, fluconazole, and griseofulvin) against the tested bacterial, yeasts and dermatophytes strains. All of them showed antimicrobial activity in the concentration range of <1.95–200 μg/mL, but with a wide variability in terms of potency and selectivity.

Regarding bacteria, the strongest inhibition was observed for E1 extract [MIC < 1.56 µg/mL against *Bacillus subtilis* (PeruMycA 6)]. Almost all of the bacterial strains were sensitive to both hydro-alcoholic extracts with MIC values lower than 62.99 μg/mL. 

The extracts were also effective in inhibiting dermatophyte growth. *A. currey* (CCF 5207) was the most sensitive fungal species to the extracts with MIC values < 6.25 μg/mL. Based on our knowledge, reports on the screening of the antimicrobial activity of *C. sativa* cv. strawberry extracts against different bacterial, yeast, and dermatophyte strains have not been published, yet. On the other hand, the present results are consistent with our previous investigations about the antimycotic effects of the water extract from the inflorescences of the industrial hemp cultivar Futura 75 [20]. 

### 3.3. Phytochemical Analysis

**Figure 1:**
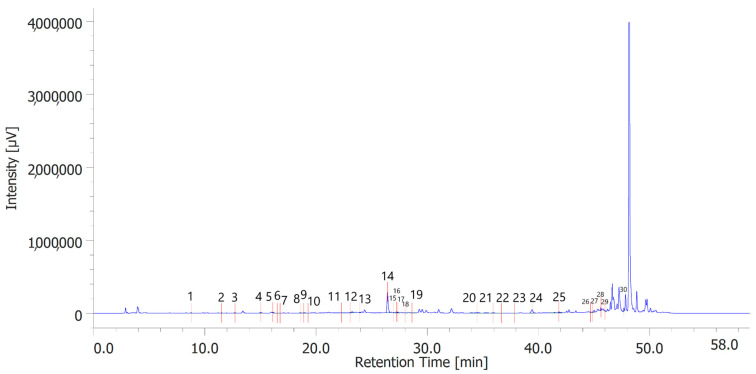
Phenolic compounds identified in the hydroalcoholic extract of hemp inflorescences. Among the compounds quantitavely determined, benzoic acid (peak #14) was the prominent.

**Figure 2:**
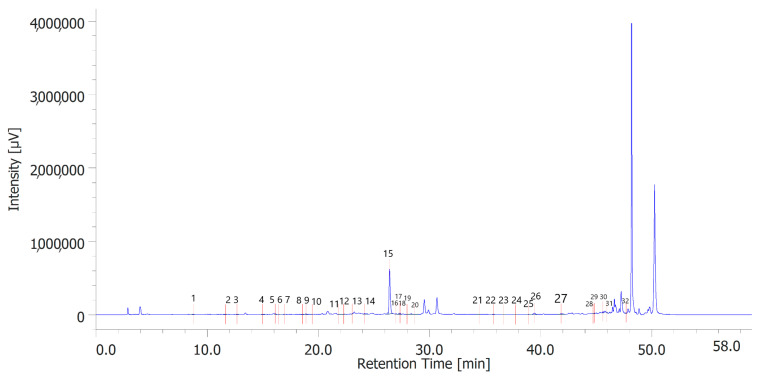
Phenolic compounds identified in the hydroalcoholic extract of hemp leaves. Among the compounds quantitatively determined, benzoic acid (peak #15) was the most prominent.

Table 6 shows the concentration of the total phenols contained (TPC) in the hydroalcoholic extracts of the leaves and inflorescences of *C. sativa* cv. strawberry. Total phenols data are expressed as % means ± SD referred to gallic acid equivalents. Phenolic compounds are an important class of active biological compounds synthesized by hemp. Their biological function is related to their antioxidant activity for the protection of important cellular structures such as membranes, structural proteins, enzymes, and cell membrane lipids. Different phenolic compounds have been identified in polar extracts from hemp inflorescences, including gallic acid, catechin, vanillic acid and rutin, and a close relationship between antioxidant activity and the content of phenolic matter [20]. In the present study, antiradical effects were measured via ABTS, DPPH, and FRAP assays (Table 7), as well. The EC_50_ values related to these assays reflected the MIC values observed against bacterial and fungal strains, thus further indicating the content of phenolics in the extracts as responsible, albeit in part, for the observed antioxidant and antimicrobial effects [35,36]. In order to improve our knowledge on the phytochemical composition of the extracts, liquid chromatography analyses were conducted (Figure 1 and Figure 2). A total of 30 compounds were identified and quantified by comparison with a pure standard. The list of the identified compounds is included in Table 3. Among the identified phytochemicals, benzoic acid was the prominent compound, in both the inflorescence (peak #14: 766.75 µg/mL) and leaf (peak # 15: 1683.43 µg/mL) extracts. This is also consistent with our recent study indicating benzoic acid as one of the prominent phytochemicals in the polar extracts from the pollen collected by male hemp inflorescences [16].

The presence of significant amounts of benzoic acid in the extracts can also explain the observed antimicrobial properties against bacteria and fungi.

Indeed, benzoic acid and its derivatives have long been used as food preservatives [37]. They are effective as antimicrobials against a wide number of microbial strains, and this can be related to their capability to penetrate the phospholipid bilayer, and disrupt phospholipids’ interactions, thus leading to membrane disintegration.

**Figure 3:**
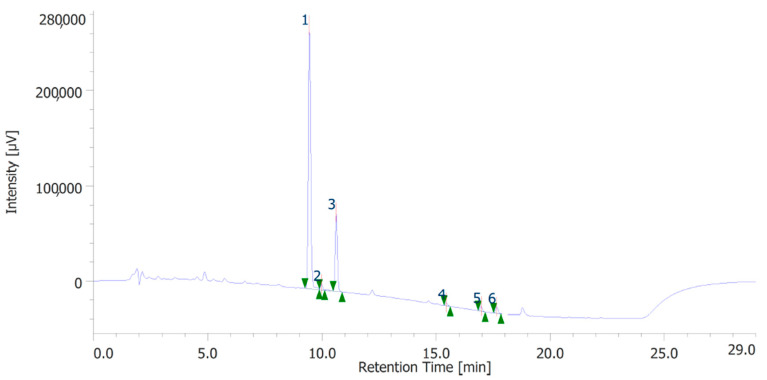
Terphenolic compounds identified in the hydroalcoholic extract of hemp inflorescences. Among the compounds quantitatively determined, cannabidiolic acid (peak #1: 45.99 µg/mL) and cannabidiol (peak #3: 23.55 µg/mL) were the most prominent.

**Figure 4:**
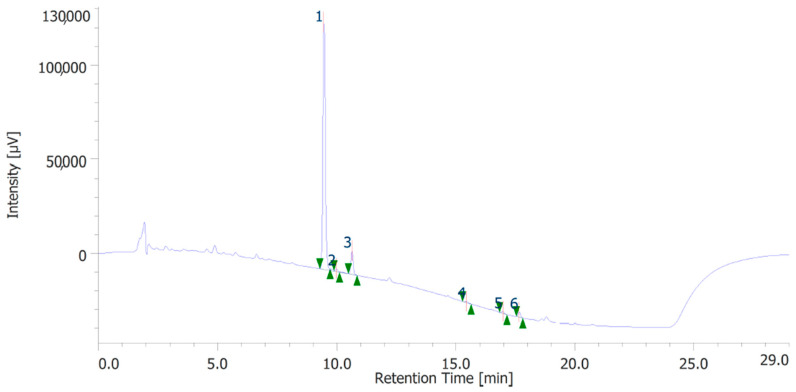
Terphenolic compounds identified in the hydroalcoholic extract of hemp leaves. Among the compounds quantitatively determined, cannabidiolic acid (peak #1: 21.71 µg/mL) and cannabidiol (peak #3: 3.52 µg/mL) were the most prominent.

The extracts were also quantitatively analyzed for the content of terpenophenols, as reported in Figure 3 and Figure 4. Both the leaves and inflorescences showed a higher content of cannabidiolic acid (CBDA, peak #1) and cannabidiol (CBD, peak # 3) compared to the other terpenophenols, whose list is reported in Table S4. Specifically, the inflorescences were richer in CBDA and CBD than the leaves. The respective concentrations of terpenophenols are indicated in the legends of Figure 3 and Figure 4.

Although these phytochemicals are present at concentrations quite lower compared with benzoic acid, we cannot exclude that CBDA and CBD may contribute to the intrinsic scavenging/reducing [10] and antimicrobial properties [38] demonstrated by the extracts.

Additionally, their presence may also suggest the extracts’ putative protective effects [16] which deserve further investigation.

## 4. Conclusions

As widely reported in the literature, *C. sativa* is a valuable crop from which multiple extracts and bioactive compounds can be obtained for innovative applications of this traditional botany resource [17]. The present study focused on the properties of different types of extracts from the strawberry hemp cultivar, with regards to phenolic composition, antioxidant and antimicrobial effects. From the outcomes highlighted above, the extracts derived from inflorescences seem to have greater biological activity than those derived from leaves, probably due to the fact that a greater number of different trichomes are present in the inflorescences and therefore there is greater and more differentiated production of active metabolites. This aspect, however, needs more in-depth studies.

The results show that this crop has antimicrobial and antioxidant activities so there are promising translational potentials in different fields. These effects could be related, albeit partially, to the total content of phenolic compounds. With specific regard to the antimicrobial effects, the higher concentrations of benzoic acid in the extracts, compared with the other phenolics, could play a pivotal role in the observed antimicrobial effects [39]. The antioxidant and antimicrobial effects of hemp extracts could be also mediated, at least in part, by the main terpenophenolics identified and quantified, namely cannabidiolic acid and cannabidiol [10,38]. Although further studies still need to increase our knowledge on the chemical composition, extracts prepared from this cultivar might be considered for the development of innovative antioxidant and antimicrobial products in the food and pharmaceutical industries. This would further improve the whole productive chain of industrial hemp.

## Figures and Tables

**Table 1:** *Cannabis sativa* L. cultivar strawberry extracts.

Sample ID	Plant Material	Type of Extract	Concentration (g/10 mL)
E1	Inflorescences	Hydroalcoholic	0.2125
E2	Leaves	Hydroalcoholic	0.1699

**Table 2:** Comparison between the sample of *C. sativa* cv. strawberry and the sequences present on GenBank.

Correspondence with Genbank seq.	% Identity	Base Pair	Accession no.
*Cannabis sativa*	100	153,945	KY084475.1
*Cannabis sativa cultivar* Dagestani	100	153,867	KR779995.1
*Cannabis sativa subsp. sativa cultivar Cheungsam*	100	153,848	KR184827.1
*Cannabis sativa cultivar* Yoruba	100	153,854	NC_027223.1
*Cannabis sativa cultivar* Carmagnola	100	153,871	NC_026562.1
*Cannabis sativa*	100	153,849	OM479429.1
*Cannabis sativa*	100	780	AF501598.1
*Cannabis sativa*	100	750	AY958392.1
*Cannabis sativa*	100	687	KF250352.1
*Cannabis sativa*	100	681	AY958387.1
*Cannabis sativa*	99.86%	153,910	MH118118.1
*Cannabis sativa*	99.86%	127,897	KY419963.1
*Cannabis sativa*	99.86%	153,927	OK523376.1
*Cannabis sativa*	99.86%	153,873	MT721158.1
*Cannabis sativa cultivar* Yunma 7	99.86%	153,899	MW013540.1
*Cannabis sativa*	99.86%	749	AY958393.1
*Cannabis sativa*	99.86%	716	JN040359.1
*Cannabis sativa*	99.85%	680	AY958388.1
*Cannabis sativa*	99.58%	750	AJ390367.1

**Table 3:** Minimal inhibitory concentrations (MICs) of *Cannabis sativa* L. cv. strawberry extracts against bacterial strains.

	MIC (µg mL^−1^) *							
	*Escherichia*	*Escherichia*	*Escherichia*	*Bacillus*	*Pseudomonas*	*Bacillus*	*Salmonella*	*Staphylococcus*
	*coli*	*coli*	*coli*	*cereus*	*aeruginosa*	*subtilis*	*typhy*	*aureus*
	(ATCC 10536)	(PeruMycA 2)	(PeruMycA 3)	(ATCC 12826)	(ATCC 15442)	(PeruMycA 6)	(PeruMycA 7)	(ATCC 6538)
E1	4.96 (3.13–6.25)	15.74 (12.5–25)	>200	>200	39.68(25–50)	1.56 < - 1.56	>200	15.74 (12.5–25)
E2	7.87 (6.25–12.5)	39.68 (25–50)	>200	>200	62.99 (50–100)	19.84 (12.5–25)	>200	62.99 (50–100)
Ciprofloxacin (µg/mL)	31.49 (25–50)	9.92 (6.25–12.5)	79.37 (50–100)	125.99 (100–200)	125.99 (100–200)	125.99 (100–200)	79.37 (50–100)	200 - > 200

* The MIC values are reported as geometric means of three independent replicates (*n* = 3). The MIC range concentrations are reported within brackets.

**Table 4:** Minimal inhibitory concentrations (MICs) of *Cannabis sativa* L. cv. strawberry extracts against yeast strains.

	MIC (µg mL^−1^) *			
	*Candida*	*Candida*	*Candida*	*Candida*
	*tropicalis*	*albicans*	*parapsilosis*	*albicans*
	(YEPGA 6184)	(YEPGA 6379)	(YEPGA 6551)	(YEPGA 6183)
E1	>200	>200	<6.25	15.75 (12.5–25)
E2	15.75 (12.5–25)	>200	<6.25	15.75 (12.5–25)
Fluconazole (µg/mL)	2	1	4	2

* The MIC values are reported as geometric means of three independent replicates (*n* = 3). The MIC range concentrations are reported within brackets.

**Table 5:** Minimal inhibitory concentrations of *Cannabis sativa* L. cv. strawberry extracts against dermatophyte strains.

	MIC (µg mL^−1^) *							
	*Trichophyton*	*Trichophyton*	*Trichophyton*	*Arthroderma*	*Trichophyton*	*Arthroderma*	*Arthroderma*	*Arthroderma*
	*mentagrophytes*	*tonsurans*	*rubrum*	*quadrifidum*	*erinacei*	*gypseum*	*currey*	*insingulare*
	(CCF 4823)	(CCF 4834)	(CCF 4933)	(CCF 5792)	(CCF 5930)	(CCF 6261)	(CCF 5207)	(CCF 5417)
E1	39.68 (25–50)	62.99 (50–100)	62.99 (50–100)	31.49 (25–50)	39.68 (25–50)	125.99 (100–200)	<6.25	125.99 (100–200)
E2	125.99 (100–200)	79.37 (50–100)	79.37 (50–100)	125.99 (100–200)	125.99 (100–200)	158.74 (100–200)	<6.25	125.99 (100–200)
Griseofulvin (µg/mL)	2.52 (2–4)	0.198 (0.125–0.25)	1.26 (1–2)	>8	3.174 (2–4)	1.587 (1–2)	>8	>8

* The MIC values are reported as geometric means of three independent replicates (*n* = 3). The MIC range concentrations are reported within brackets.

**Table 6:** Total phenolics compounds contained in *C. sativa* cv. strawberry extracts.

Sample	GAE	±SD
E1	14.97	1.51
E2	13.79	1.39

**Table 7:** Antiradical activity of the tested *C. sativa* extracts.

	DPPH Test		ABTS Test		FRAP Test
Sample	EC_50_ μg/mL	Trolox Equivalents	EC_50_ μg/mL	Trolox Equivalents	Trolox Equivalents
E1	73 ± 2	11.45 ± 0.39	2 ± 0.04	1.13 ± 0.02	95 ± 50
E2	67 ± 5	10.31 ± 0.85	2 ± 0.1	1.29 ± 0.06	76 ± 12

## Data Availability

The original data are available from the corresponding author.

## Supplementary Materials

The following supporting information can be downloaded at: https://www.mdpi.com/article/10.3390/antiox12020219/s1. High-performance liquid chromatography (HPLC) analysis of phenolic and terpenophenolic compounds; Table S1: Gradient elution condition; Table S2: Retention times and wavelength of quantification of the standards used to identify and quantify phenolics in the extracts.; Table S3: Gradient elution of HPLC-DAD; Table S4: Retention times and wavelength of the quantification of the standards used to identify and quantify the terpenophenolics in the extracts.
