# Supplementary material for: Comparative Investigation of Antimicrobial and Antioxidant Effects of the Extracts from the Inflorescences and Leaves of the Cannabis sativa L. cv. strawberry
Source: Antioxidants (Basel). 2023 Jan 18;12(2):219. doi: 10.3390/antiox12020219 (PMC9951856; doi:10.3390/antiox12020219)
Supplement: Supplementary file 1 [file antioxidants-12-00219-s001.zip › antioxidants-2124879-supplementary.pdf]

High performance liquid chromatography (HPLC) analysis of phenolic and terpenophenolic compounds

The HPLC apparatus consisted of a two PU-2080 PLUS chromatographic pump, a DG-2080-54 line degasser, a mix-2080-32 mixer, diode array (DAD), an AS-2057 PLUS autosampler and a CO-2060 PLUS column thermostat (all from Jasco, Tokyo, Japan). Integration was performed by ChromNAV2 Chromatography software. Before injecting in the HPLC apparatus, hemp extracts were centrifuged at 3500× g for 15 min, and supernatant diluted at 1 mg/mL for the identification and quantitative analysis of phenolic and terpenophenolic compounds through comparison with respective standards.

Regarding the phenol quantitative determination, the extracts were analyzed using a reversed-phase HPLC-DAD in gradient elution mode (Table S1). The separation was conducted within 60 min of the chromatographic run, starting from the following separation conditions: 97 % water with 0.1 % formic acid, 3 % methanol with 0.1 % formic acid. The separation was performed on an Infinity lab Poroshell 120-SB reverse phase column (C18, 150 × 4.6 mm i.d., 2.7 μm; Agilent, Santa Clara, CA, USA). Column temperature was set at 30 °C. Quantitative determination of phenolic compounds was performed via a DAD detector. Quantification was done through 7-point calibration curves, with linearity coefficients (R2) > 0.999, in the concentration range 2–140 μg/mL. All standards were purchased from Sigma Aldrich (Milan, Italy), and have a purity ≥ 95 %. The limits of detection were lower than 1 μg/mL for all assayed analytes (Table S2). The area under the curve from HPLC chromatograms was used to quantify the analyte concentrations in the extract. The wavelength of quantification with the Photo Diode Array Detector was set at 254 nm.

The quantitative determination of terpenophenols was conducted using a reversed-phase HPLC–UV-MS in gradient elution mode. The separation was conducted within 30 min, starting from the following conditions: 0.007% formic acid, 7% water, 93% acetonitrile. The details about gradient are listed in Table S3. The separation was performed on an Infinity lab Poroshell 120 reverse phase column (C18, 150 mm × 4.6 mm i.d., 2.7 μm) (Agilent Santa Clara, CA, USA). Column temperature was set at 30 °C.

Quantitative determination of terpenophenolic compounds (Table S4) was performed via DAD detector at 230 nm wavelength. Quantification was performed through 7-point calibration curves, with linearity coefficients (R2) > 0.99, in the concentration range 2–160 μg/mL. The area under the curve from HPLC chromatograms was used to quantify the terpenophenol concentration in the extract. The limits of detection were lower than 0.9 μg/mL for all assayed analytes.

Table S1. Gradient Elution Conditions

TIME (Min.)	COMPOSITION A% (Water+Formic acid 0.1%)	COMPOSITION B% (Methanol+Formic acid 0.1%)	FLOW (mL/min)
1.00	97.0	3.0	0.600
5.00	77.0	23.0	0.600
12.00	73.0	27.0	0.600
18.00	57.0	43.0	0.600
25.00	52.0	48.0	0.600
32.00	50.0	50.0	0.600
34.00	50.0	50.0	0.600
37.00	35.0	65.0	0.600
40.00	5.0	95.0	0.600
47.00	5.0	95.0	0.600
48.00	97.0	3.0	0.600
60.00	97.0	3.0	0.600

Table S2. Retention times and wavelength of quantification of the standards used to identify and quantify phenolics in the extracts.

	Standard	Wavelength (nm)	Retention Time (min)
1	Gallic acid	254	8.770
2	3-Hydroxytyrosol	254	11.483
3	Caftaric acid	254	12.693
4	Catechin	254	15.007
5	4-Hydroxybenzoic acid	254	16.117
6	Loganic acid	254	16.503
7	Chlorogenic acid	254	16.750
8	Vanillic acid	254	18.567
9	Caffeic acid	254	18.887
10	Epicatechin	254	19.273
11	Syringaldehyde	254	21.833
12	Chicoric acid	254	22.283
13	Coumaric acid	254	23.0.43
14	Ferulic acid	254	24.133
15	Benzoic acid	254	26.407
16	Hyperoside	254	26.970
17	Rutin	254	27.263
18	Resveratrol	254	28.787
19	Isoquercetin	254	27.347
20	Resveratrol	254	27.980
21	Rosmarinic acid	254	28.633
22	t-Cinnamic acid	254	34.453
23	Quercetin	254	35.747
24	Naringenin	254	36.647
25	2,3-Dimethylbenzoic acid	254	37.743
26	Luteolin	254	38.907
27	Hesperitin	254	39.420
28	Kaempferol	254	41.827
29	Carvacrol	254	44.717
30	Thymol	254	44.837
31	Flavone	254	45.607
32	3-Hydroxyflavone	254	45.940
33	Emodin	254	47.683

**Table S3 Gradient elution of HPLC-DAD**			
**Time (min.**	**Flow (ml/min)**	**%A**	**%B**
0	0.750	32.5	67.5
0.5	0.750	32.5	67.5
14	0.750	7	93
22	0.750	7	93
22.1	1.05	32.5	67.5
28	1.05	32.5	67.5
28.1	0.750	32.5	67.5
30	0.750	32.5	67.5

Table S4. Retention times and wavelength of quantification of the standards used to identify and quantify terpenophenolics in the extracts.

	**Standard**	**Wavelenght *(nm)***	**Retention time (min)**
1	CBDA	*230*	9.433
2	CBGA	230	9.967
3	CBD	230	10.600
4	THC-d3	230	15.433
5	CBC	230	16.967
6	THCA	230	17.633
